# Association of Funding and Meal Preparation Time With Nutritional Quality of Meals of Supplemental Nutritional Assistance Program Recipients

**DOI:** 10.1001/jamanetworkopen.2021.14701

**Published:** 2021-06-24

**Authors:** Matt Olfat, Barbara A. Laraia, Anil J. Aswani

**Affiliations:** 1 Industrial Engineering and Operations Research, University of California, Berkeley; 2 Now with Citadel LLC, Chicago, Illinois; 3 School of Public Health, University of California, Berkeley

## Abstract

**Importance:**

The Supplemental Nutrition Assistance Program (SNAP) is a federal program that provides food-purchasing assistance to low-income people; however, its current design does not account for the time availability of SNAP recipients to prepare meals.

**Objective:**

To evaluate the association of the availability of funding for food purchases and time for meal preparation with the nutritional quality of meals of SNAP recipients.

**Design, Setting, and Participants:**

This study used decision analytical modeling to evaluate the nutritional quality of meals of SNAP recipients. The model was developed from February 6, 2017, to December 12, 2020, using data from 2017 and is based on discrete optimization. The model describes food and grocery purchasing, in-home meal preparation, and meal plan choices of a family of SNAP participants (2 adults and 2 children) while considering food preferences, meal preparation time, and food costs. The model assumes food preferences match the foods typically purchased by SNAP households. Costs of food ingredients and prepared foods are taken from a single zip code.

**Exposures:**

Time availability and total amount and type of funding were varied. Allowing prepared delicatessen foods and disallowing frozen prepared foods for purchase using SNAP funds were considered.

**Main Outcomes and Measures:**

The primary outcome was the number of home-cooked meals and the amounts of fruits, vegetables, protein, sodium, sugar, and fiber consumed from generated meal plans. Amounts were evaluated as a percentage of the quantity recommended by established dietary guidelines.

**Results:**

Increased time availability was associated with increases in the percentage of home-cooked meals and servings of fruits/vegetables and decreased sodium consumption. Higher levels of funding were associated with increased consumption of fiber, fruits/vegetables, protein, sodium, and sugar. With 20 min/d of cooking time, $400/mo of SNAP benefits, and $100/mo of self-funding, the meal plan had a mean (SE) of 20.1% (0.3%) of meals home cooked, 0.5 (<0.1) servings/d per person of fruits/vegetables, 100.3% (0.6%) of daily recommended protein per person, 115.1% (0.8%) of daily recommended sodium per person, 241.8% (1.0%) of daily recommended sugar per person, and 31.2% (0.3%) of daily recommended fiber per person. With 20 min/d of cooking time, $400/mo of SNAP benefits, and $600/mo of self-funding, the meal plan had a mean (SE) of 23.9% (1.0%) of meals home cooked, 2.8 (0.1) servings/d per person of fruits/vegetables, 134.9% (1.6%) of daily recommended protein per person, 200.9% (3.1%) of daily recommended sodium per person, 295.1% (3.1%) of daily recommended sugar per person, and 90.1% (1.0%) of daily recommended fiber per person. With 60 min/d of cooking time, $400/mo of SNAP benefits, and $100/mo of self-funding, the meal plan had a mean (SE) of 52.7% (0.9%) of meals home cooked, 1.4 (<0.1) servings/d per person of fruits/vegetables, 109.0% (1.1%) of daily recommended protein per person, 108.7% (1.0%) of daily recommended sodium per person, 298.6% (2.0%) of daily recommended sugar per person, and 38.8% (0.4%) of daily recommended fiber per person. With 60 min/d of cooking time, $400/mo of SNAP benefits, and $600/mo of self-funding, the meal plan had a mean (SE) of 42.8% (1.2%) meals home cooked, 4.3 (0.1) servings/d per person of fruits/vegetables, 144.4% (1.8%) of daily recommended protein per person, 165.2% (2.8%) of daily recommended sodium per person, 322.4% (2.4%) of daily recommended sugar per person, and 91.0% (0.9%) of daily recommended fiber per person.

**Conclusions and Relevance:**

In this decision analytical model, meal preparation time was associated with the ability of SNAP recipient families to consume nutritious meals, suggesting that increased funding alone may be insufficient for improving the nutritional profiles of SNAP recipients. Given the current US food supply, governmental interventions that provide the equivalence in increased time availability to achieve nutritious meals may be needed.

## Key Points

**Question:**

Is there an association between the nutritional quality of Supplemental Nutrition Assistance Program (SNAP) meals and changes in availability of food purchase funding and time for meal preparation?

**Findings:**

This decision analytical modeling study found that increased time availability was associated with an increased percentage of home-cooked meals, increased consumption of fruits and vegetables, and reduced consumption of sodium among SNAP recipients.

**Meaning:**

The findings of this study suggest that increased funding alone may be insufficient for improving the nutritional profiles of SNAP recipients and that increased time availability may be required to achieve nutritious meals.

## Teaser

This study uses decision analytical modeling to evaluate the association of food purchase funding and meal preparation time with the nutritional quality of meals consumed by recipients of the Supplemental Nutrition Assistance Program.

## Introduction

The Supplemental Nutrition Assistance Program (SNAP) is a federal nutrition assistance program that aids more than 20 million households by giving participants a mean of $125 in benefits per month.^1^ Although SNAP reduces food insecurity,^2^ participants consume fewer fruits, vegetables, and nuts; get more calories from sweetened drinks and processed meats; and have higher levels of obesity and type 2 diabetes than higher-income US residents.^3,4,5^ These disparities are increased by societal trends: away-from-home meals rose from 20% of all food spending in 1961^6^ to 44% in 2017,^7^ and families have shifted to 2-worker households.^8^ Increasing the proportion of meals that are home cooked is important because cooking leads to more nutritious meals and increased fruit and vegetable consumption,^9,10,11,12^ and higher levels of fruit and vegetable consumption significantly reduce all-cause cancer and mortality.^13,14^

As a result, recent interest has been shown in revising the design of SNAP to improve the quality of food consumed.^15,16,17^ One proposal is to restrict foods that can be purchased using SNAP benefits, such as banning the purchase of sugary drinks like soda.^18,19^ A related proposal is to allow only items from a specific list to be purchased with SNAP funds.^20^ However, restricting food purchases using SNAP has met opposition due to concerns regarding fairness.^21,22^ Other proposals include making hot prepared food eligible for purchase with SNAP benefits^23^ and providing discounts or rebates on fruits and vegetables purchased via SNAP,^24^ although the success of such rebate schemes has been limited.

SNAP’s current design originates from the Thrifty Food Plan (TFP), which is based on decision analytical modeling using continuous optimization.^25,26^ This food plan ensures adequate nutrient intake, enough food servings, and feasibility in terms of cost and adjusts for the dietary preferences of SNAP participants. However, the TFP was developed under the assumption that households can devote unlimited time per day to meal preparation and did not consider how cooking time affects dietary choices and outcomes, and the resulting TFP requires approximately 2 hours per day of meal preparation time.^27^ This time investment is problematic because low-income families and SNAP families spend a mean of approximately 40 and 50 minutes, respectively, on meal preparation.^28^ Households have limited time availability for cooking,^29,30,31^ especially those families without the ability to “buy time” through childcare and household services.^32,33^ More realistic SNAP food plans are needed.

Decision analytical modeling is a valuable means of informing changes to SNAP. This approach has been used to address challenges in health care^34^ and food supply chains^35^ by predicting a range of outcomes under complex conditions and options. In the present study, we performed decision analytical modeling using a discrete optimization approach to understand how the nutritional quality of meals consumed by SNAP participants changes as more money or more time becomes available. This consideration is important because analysis of outcomes under more realistic assumptions is needed to better position SNAP in a changing food landscape. Unlike the TFP, our model takes into consideration meal preparation time as well as meal consumption habits and the cost of store-bought meals and ingredients. Our model accounts for meal preparation time by relating the discrete amounts of purchased ingredients in a food plan to specific recipes for food dishes. We used this model to analyze the sensitivity of the nutritional profile of SNAP participants to primary food choice factors in a way that is not possible through solely empirical studies and then make relevant recommendations.

This study considers 2 questions. First, how does the nutritional quality of meals of SNAP beneficiaries change in association with varying funding for food purchases and time availability for meal preparation? Our hypothesis was that the nutritional quality of meals would be more strongly associated with time availability than funding amount. Second, would changing the rules for SNAP to allow purchase of prepared deli foods (eg, rotisserie chicken, deli sandwiches, hot soups, deli salads) or to disallow purchase of prepared frozen foods (eg, frozen pizza, frozen lasagna) be associated with the nutritional quality of meals consumed by SNAP recipients? Our hypothesis was that allowing purchase of deli foods and disallowing purchase of frozen foods using SNAP funds would be associated with an improvement in the nutritional quality of meals.

## Methods

This study followed the Consolidated Health Economic Evaluation Reporting Standards (CHEERS) reporting guideline for decision analytical models.^36^ The University of California, Berkeley, institutional review board waived review of the study because it does not constitute human subject research according to the relevant regulations.

### Decision Analytical Model

Our decision analytical model simulates the food purchase choices and meal plan of an average SNAP-enrolled family of 4 during a month, which is the period between SNAP disbursements. We consider a family of 4 because this is the size of the reference family for which the TFP was developed.^26^ The parameters and assumptions of our model are provided in Table 1, and the mathematical formulation of our model and the data sources used for calibration are described in the eMethods in the Supplement. The model was developed from February 6, 2017, to December 12, 2020, and it uses prices from 2017. We capture preferences by calibrating the model to a ranking of foods typically purchased by SNAP households as identified by transaction data of 26.5 million SNAP households from a large grocery retailer.^37^ Our model considers constraints on the amount of SNAP benefits, household funds available for food, and time availability to prepare meals. The model considers meal preparation time by matching purchased ingredients to specific food recipes and then summing the total times for each recipe. In addition to purchasing raw ingredients, our model also includes the purchase of prepared frozen, deli, and restaurant foods.

**Table 1.** Parameters of Decision Analytical Model

Parameter	Assumptions
**Inputs **	
Ranking of 30 food groups by SNAP household expenditures^37^	Scores with range of 30 to 1 assigned to ingredients from food groups with highest to lowest expenditures, respectively
Costs of 400 raw ingredients, 7 sweetened beverages, 15 prepared cold foods (eg, cereal, pastry, or chips), 21 prepared frozen foods, and 10 prepared deli foods	Price data accessed in 2017 from Safeway, Inc’s online shopping site for the zip code 94702 (Berkeley, California)^38^
Costs of 82 restaurant-prepared fast foods	Prices for fast foods were accessed in 2017 from the Fast Food Menu Prices website^39^
Nutrient profile of raw ingredients, sweetened beverages, and prepared foods	Nutritional information accessed in 2017 from the USDA’s online Food Composition Database^40^
Recipes of 3327 foods, consisting of raw ingredient quantities and cooking times	Recipes accessed in 2017 from British Broadcasting Corporation Food online database of 11 026 recipes^41^ and list of recipes curated to select foods more commonly eaten in the US
Preference score for a recipe, sweetened beverage, or prepared food	Score is a weighted-by-proportion sum of ingredient-preference scores; on each simulation, this score is multiplied by a randomly chosen value from 0.9 to 1.1 to model family-specific variances in preferences
**Constraints**	
Total cost of purchased raw ingredients, sweetened beverages, and prepared foods is less than total available funding	Pricing is unit based, meaning there are no volume discounts for large purchase quantities
Total cost of SNAP-ineligible prepared foods is less than the amount of unrestricted personal funds	Prepared deli foods and restaurant foods are currently SNAP ineligible; association with proposal to allow deli foods and disallow frozen prepared foods was also analyzed
Total time spent preparing meals is less than time budget	Time required to prepare a food is the value given in the recipe, and zero time is required to prepare a prepared food or beverage
Quantity of purchased raw ingredients is sufficient to cook recipes selected in meal plan	Recipes are prepared without substitution or changes, but a single main ingredient (eg, fish) can be prepared in different ways by choosing different recipes (eg, recipe for baked fish or recipe for fried fish)
Any individual recipe, sweetened beverage, or prepared food is not consumed many times in 1 mo	No recipe or prepared food is consumed >5 times/mo per family member, but this limit is doubled for beverages and salty snacks
Each family member will eat ≥30 lunches, ≥30 dinners, and ≥20 breakfasts in a month	More than one-half of US residents aged <65 y do not regularly eat breakfast, with 25% of adults reporting they rarely or never eat breakfast^42^
Each family member will eat ≥1 meal of >600 calories each day	Meal plans cannot have extremely low calories because otherwise families would not survive
Ingredients, beverages, prepared foods, and recipes are selected in whole amounts	Food purchases cannot be fractions of a package/unit, and fraction of recipe cannot be prepared
**Objective and output **	
Chosen meal plan consisting of raw ingredients, sweetened beverages, prepared foods, and list of recipes	Meal plan choice maximizes preference score of selected recipes, sweetened beverages, or prepared foods based on family preference of meal plan rather than nutrition

Abbreviations: SNAP, Supplemental Nutrition Assistance Program; USDA, US Department of Agriculture.

### Comparison With TFP Model

There are several differences between our model and the model used to design the TFP.^26^ The goal of the TFP model is to construct a meal plan that fits a financial budget by choosing purchase quantities from among 58 food groups to most closely match current consumption patterns while ensuring the meal plan meets nutritional criteria, such as sufficient quantities of nutrients (eg, vitamin A), limits on total fat and sodium, and other criteria. However, the nutritional profile of the TFP does not match the nutrition of a typical family^43,44,45,46^ because the TFP model does not attempt to model the anticipated purchases and meal habits of beneficiaries. Moreover, the TFP model does not consider meal preparation time because it does not match ingredients to recipes. The resulting TFP requires approximately 2 hours per day of meal preparation time.^27^ The TFP is also based on continuous optimization because the model constructs meal plans in terms of 58 food groups rather than any specific ingredients or prepared meals. In contrast, the goal of our model is to simulate the purchasing and meal plan behaviors of SNAP recipients to match their current nutritional profile^43,44,45,46^ and current consumption patterns^37^ while ensuring that the total cost and cooking time of the meal plan meet a budget. Also, our model is based on discrete optimization.^47^ Continuous optimization allows ingredients to be purchased in fractional quantities, whereas discrete optimization forces ingredients to be purchased in whole (ie, integer-valued) quantities. Our model cannot be formulated using continuous optimization because that approach cannot properly capture the association between selecting recipes and relating their cooking time to the total time availability budget, the reason being that the cooking time for a fraction of a recipe would not equal the same fraction of the cooking time of the entire recipe.

### Outcome Measures

The metrics used to compare 2 given meal plans are the proportion of cooked meals (vs prepared meals), daily fruit and vegetable intake, and daily intake of protein, sodium, fiber, and sugar. All recommendations and baselines are reported as the total recommendation for a family of 4, consisting of a man and a woman aged 31 to 50 years and a boy and a girl aged 14 to 18 years. Therefore, a baseline of 122 g of sugar per day would imply 122 g consumed by the entire family as a group as opposed to each individual. Fruit and vegetable intake are reported in servings, wherein a serving is equivalent to 6 ounces (approximately 1 cup); this amount may be compared with the daily recommendation of servings of fruits (10 cups) and vegetables (16 cups) per the 2015-2020 Dietary Guidelines for Americans.^48^ Protein intake is reported as a percentage of the estimated average recommendation (276 g/d)^49^; sodium, as a percentage of the tolerable upper intake level (9.2 g/d)^49^; fiber, as a percentage of the estimated average recommendation (127 g/d)^49^; and the total amount of added sugar, as a percentage of the maximum recommended by the American Heart Association (122 g/d).^50^

### Analysis Conducted With Model

In each analysis, we performed 100 simulations at a given budget level and report the mean (SE) of the outcome measures. The result of each simulation differs because in each simulation, we multiply the preference score of each recipe or prepared food by a randomly chosen value from 0.9 to 1.1. This procedure modeled interfamily variations in food preferences.

The first analysis evaluates outcomes because the amounts of funding and time availability are varied across a range that is representative of most SNAP households. The maximum monthly benefit per person is $144, and the mean SNAP benefit per person is $125,^51^ which correspond to a maximum SNAP benefit of approximately $600 and mean of $500 for a family of 4. Because the average household spends approximately $740 each month on food,^52^ our analysis considers a range of household funds from $100 to $600 (incrementing by $100) so that a total budget of $700 to $800 is within the middle of the range of total budgets considered. Time spent on food preparation, cooking, and cleaning has declined.^53,54,55^ In the US, the mean time for cooking was estimated at 61 minutes in 1985, declining to 42 minutes in 2010.^54^ In 2016, US residents spent a mean of 37 minutes on cooking per day.^56^ A greater percentage of employed individuals spend 1 hour or less, whereas wealthier households report spending 2 or more hours on cooking and food preparation.^55^ Low-income families and SNAP families spend a mean of approximately 40 and 50 minutes, respectively, on meal preparation.^28^ Time-use surveys ask for total time spent on cooking and do not ask respondents for their time availability for cooking. Thus, our analysis used the above total time spent cooking and considered a range of meal preparation time availability values, which are similar in magnitude to total time spent cooking, from 20 to 60 minutes (in 10-minute increments).

The second analysis conducted with our model evaluated outcomes if the allowability of SNAP purchases are changed. Prepared deli foods (eg, rotisserie chicken, deli sandwiches, hot soups, deli salads) are currently not eligible for purchase with SNAP benefits, whereas many frozen prepared foods are eligible. We evaluated the meal plans resulting from the corresponding changes to SNAP in which deli foods are made eligible or frozen prepared foods are made ineligible. We considered a range of time availability and funding values similar to those of the first analysis.

## Results

The Figure summarizes the nutritional quality of meal plans, as predicted by our model, for a family of 4 with a fixed $400/mo in SNAP benefits and varied time availability for meal preparation and amount of self-funding. The numerical values shown in this Figure are found in the eTable in the Supplement. Our model is based on discrete optimization, which makes the simulation outputs nonlinear because the model allows a SNAP family to make a discrete change (as time availability and funding are varied) in the specific recipes and foods that constitute their meal plan. This nonlinearity of model output can manifest itself as the crossing of curves, as shown in the Figure, C. With 20 min/d of cooking time, $400/mo of SNAP benefits, and $100/mo of self-funding, the meal plan had a mean (SE) of 20.1% (0.3%) of meals home cooked, 0.5 (<0.1) servings/d per person of fruits/vegetables, 100.3% (0.6%) of daily recommended protein per person, 115.1% ( 0.8%) of daily recommended sodium per person, 241.8% (1.0%) of daily recommended sugar per person, and 31.2% (0.3%) of daily recommended fiber per person. With 60 min/d of cooking time, $400/mo of SNAP benefits, and $100/mo of self-funding, the meal plan had a mean (SE) of 52.7% (0.9%) of meals home cooked, 1.4 (<0.1) servings/d per person of fruits/vegetables, 109.0% (1.1%) of daily recommended protein per person, 108.7% (1.0%) of daily recommended sodium per person, 298.6% (2.0%) of daily recommended sugar per person, and 38.8% (0.4%) of daily recommended fiber per person.

**Figure.**
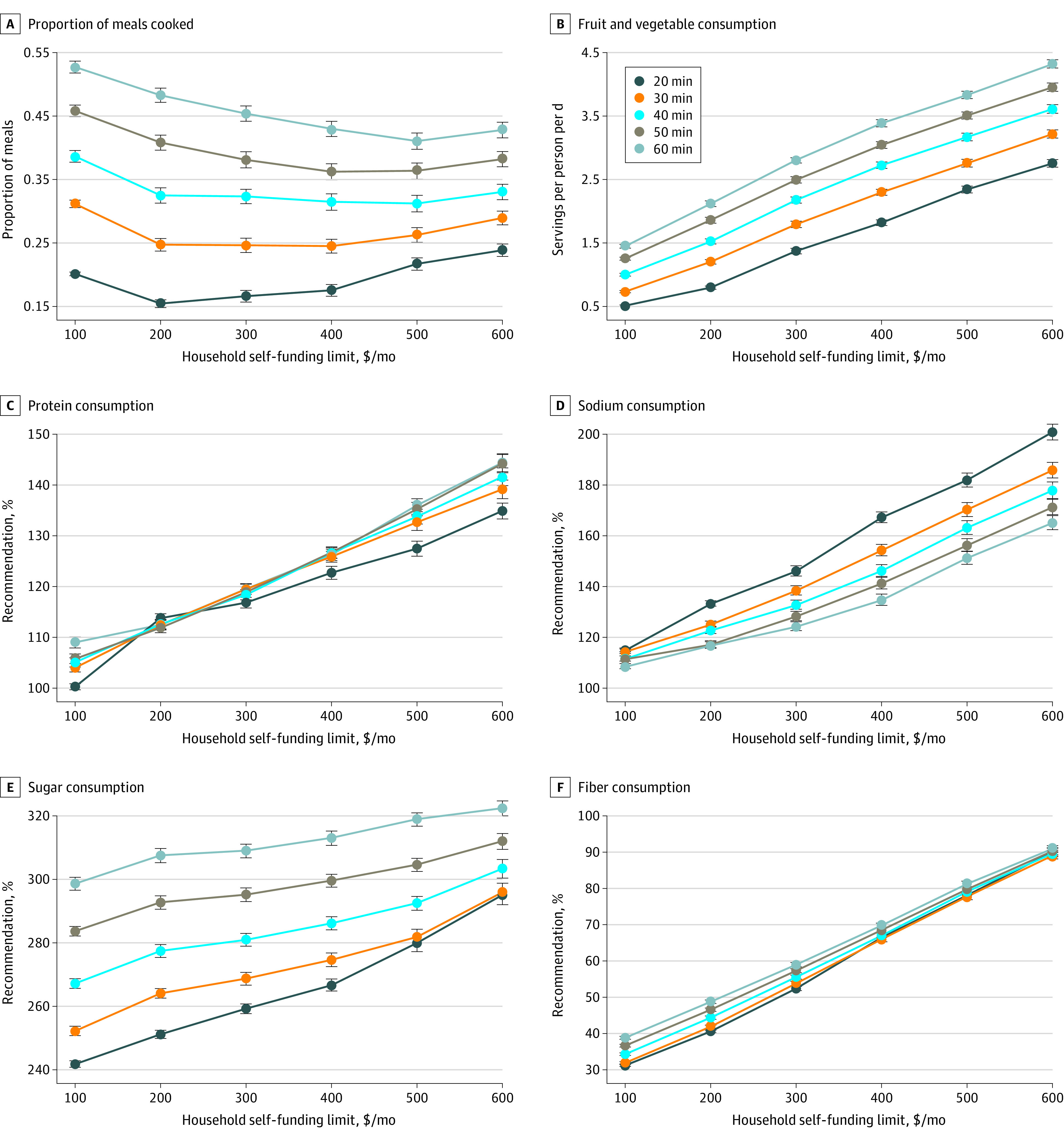
Nutritional Intake of a Typical Family of 4 Under Several Financial and Time Availability Scenarios Under Existing Supplemental Nutrition Assistance Program (SNAP) Policies SNAP funding for each scenario was $400/mo. Recommended protein consumption is 276 g/d or a mean of 29 g/d per person; recommended sodium consumption, 9.2 g/d or a mean of 2.3 g/d per person; recommended sugar consumption, 122 g/d or a mean of 30.5 g/d per person; and recommended fiber consumption, 127 g/d or a mean of 31.75 g/d per person.

The Figure, A, shows that there was an association between an increase in the proportion of home-cooked meals and increased time availability. With $200/mo of self-funding at 20 min/d of cooking time, the mean (SE) proportion of home-cooked meals was 15.5% (0.6%); at 60 min/d of cooking time, it was 48.3% (1.1%). In contrast, there was no association between a higher household spending limit and an increase in the proportion of home-cooked meals. With $600/mo of self-funding at 20 min/d of cooking time, the mean (SE) proportion of home-cooked meals was 23.9% (1.0%); at 60 min/d of cooking time, it was 42.8% (1.2%).

The Figure, B, shows that more time availability and more household spending was associated with an increase in the mean servings of fruits and vegetables. With $200/mo of self-funding at 20 min/d of cooking time, the meal plan had a mean (SE) of 0.8 (<0.1) servings/d per person of fruits/vegetables; at 60 min/d of cooking time, it had a mean (SE) of 2.1 (<0.1) servings/d per person. With $600/mo of self-funding at 20 min/d of cooking time, the meal plan had a mean (SE) of 2.8 (0.1) servings/d per person of fruits/vegetables; at 60 min/d of cooking time, the meal plan had a mean (SE) of 4.3 (0.1) servings/d per person.

The Figure, C, shows that the association between more household spending and increased percentage of recommended protein consumption was stronger than that for more time availability. The lowest mean (SE) percentage of recommended protein consumption was 100.3% (0.6%) and occurred with 20 min/d of cooking time and $100/mo of self-funding, and the highest mean (SE) percentage of recommended protein consumption was 144.4% (1.8%) and occurred with 60 min/d of cooking time and $600/mo of self-funding. For 20 min/d of cooking time and $600/mo of self-funding, the mean (SE) of daily recommended protein per person was 134.9% (1.6%).

The Figure, D, shows that more time availability, but not more household spending, was associated with a decrease in the percentage of recommended sodium consumption. With $200/mo of self-funding at 20 min/d of cooking time, the meal plan had a mean (SE) of 113.4% (1.1%) of daily recommended sodium per person; at 60 min/d of cooking time, it had a mean (SE) of 117.0% (1.3%) of daily recommended sodium per person. With $600/mo of self-funding at 20 min/d of cooking time, the meal plan had a mean (SE) of 200.9% (3.1%) of daily recommended sodium per person; at 60 min/d of cooking time, it had a mean (SE) of 165.2% (2.8%) of daily recommended sodium per person.

The Figure, E, shows that more time availability and more household spending were both associated with an increase in the percentage of recommended sugar consumption. With $200/mo of self-funding and at 20 min/d of cooking time, the meal plan had a mean (SE) of 251.2% (1.2%) of daily recommended sugar per person; at 60 min/d of cooking time, it had a mean (SE) of 307.5% (2.2%) of daily recommended sugar per person. With $600/mo of self funding and at 20 min/d of cooking time, the meal plan had a mean (SE) of 295.1% (3.1%) of daily recommended sugar per person; at 60 min/d of cooking time, it had a mean (SE) of 322.4% (2.4%) of daily recommended sugar per person.

The Figure, F, shows that the association between more household spending and increased percentage of recommended fiber consumption was stronger than that for more time availability. With $200/mo of self-funding at 20 min/d of cooking time, the meal plan had a mean (SE) of 40.6% (0.4%) of daily recommended fiber per person; at 60 min/d of cooking time, it had a mean (SE) of 48.8% (0.5%) of recommended fiber/d per person. With $600/mo of self-funding at 20 min/d of cooking time, the meal plan had a mean (SE) of 90.1% (1.0%) of daily recommended fiber per person; at 60 min/d of cooking time, it had a mean (SE) of 91.0% (0.9%) of daily recommended fiber per person.

As described in the Methods section, we also explored the potential effects of including deli foods as food eligible for purchase with SNAP benefits, with the aim of providing an alternative for frozen prepared foods. Simulation results for our model under both conditions—that is, when SNAP funds can and cannot be spent on deli foods—are summarized in Table 2 and Table 3, respectively. (Note that total spending always amounts to $800 in both cases, so the results reported in Tables 2 and 3 are best compared with the points in the Figure with $400 of self-funding.) Comparing these results shows that allowing SNAP funds to be spent on deli foods is not associated with changes in the nutritional quality of meal plans relative to meal plans that do not allow SNAP funds to be spent on deli foods. For example, in the scenario with 40 min/d of cooking time, $400/mo of SNAP benefits, and $400/mo of self-funding, the meal plan for which SNAP funds cannot be spent on deli foods had a mean (SE) of 2.5 (0.1) servings/d per person of fruits/vegetables, 130.5% (1.3%) of daily recommended protein per person, 155.6% (2.5%) of daily recommended sodium per person, 285.7% (2.0%) of daily recommended sugar per person, and 67.6% (0.8%) of daily recommended fiber per person. The meal plan for which SNAP funds can be spent on deli foods had a mean (SE) of 2.7 (0.1) servings/d per person of fruits/vegetables, 126.2% (1.2%) of daily recommended protein per person, 151.6% (2.6%) of daily recommended sodium per person, 287.0% (1.7%) of daily recommended sugar per person, and 67.9% (0.7%) of daily recommended fiber per person.

**Table 2.** Nutritional Intake of a Family of 4 by Funding and Time Availability Under Existing SNAP Policies

Time, min/d	SNAP funding, $/mo	Self-funding, $/mo	No. of servings of fruits/vegetables per day per person, mean (SE)	Recommended consumption, mean (SE), %			
				Protein	Sodium	Sugar	Fiber
20	700	100	1.7 (<0.1)	126.9 (1.4)	173.5 (2.3)	267.3 (1.7)	66.2 (0.8)
20	600	200	1.7 (0.1)	126.3 (1.4)	174.3 (2.3)	265.3 (1.7)	66.0 (0.8)
20	500	300	1.7 (0.1)	126.3 (1.4)	174.3 (2.3)	265.3 (1.7)	66.0 (0.8)
20	400	400	1.7 (0.1)	126.3 (1.4)	174.3 (2.3)	265.3 (1.7)	66.0 (0.8)
20	300	500	1.7 (0.1)	126.3 (1.4)	174.3 (2.3)	265.3 (1.7)	66.0 (0.8)
30	700	100	2.1 (0.1)	129.7 (1.3)	163.3 (2.4)	275.8 (1.9)	66.9 (0.7)
30	600	200	2.2 (0.1)	129.2 (1.3)	163.0 (2.5)	275.3 (1.9)	66.6 (0.8)
30	500	300	2.2 (0.1)	129.2 (1.3)	163.0 (2.5)	275.3 (1.9)	66.6 (0.8)
30	400	400	2.2 (0.1)	129.2 (1.3)	163.0 (2.5)	275.3 (1.9)	66.6 (0.8)
30	300	500	2.2 (0.1)	129.2 (1.3)	163.0 (2.5)	275.3 (1.9)	66.6 (0.8)
40	700	100	2.5 (0.1)	130.8 (1.3)	155.4 (2.4)	285.6 (2.0)	67.6 (0.8)
40	600	200	2.5 (0.1)	130.5 (1.3)	155.6 (2.5)	285.7 (2.0)	67.6 (0.8)
40	500	300	2.5 (0.1)	130.5 (1.3)	155.6 (2.5)	285.7 (2.0)	67.6 (0.8)
40	400	400	2.5 (0.1)	130.5 (1.3)	155.6 (2.5)	285.7 (2.0)	67.6 (0.8)
40	300	500	2.5 (0.1)	130.5 (1.3)	155.6 (2.5)	285.7 (2.0)	67.6 (0.8)
50	700	100	2.9 (0.1)	130.6 (1.3)	149.2 (2.3)	298.1 (2.0)	69.1 (0.7)
50	600	200	2.9 (0.1)	130.4 (1.2)	149.0 (2.4)	298.5 (2.0)	69.1 (0.7)
50	500	300	2.9 (0.1)	130.4 (1.2)	149.0 (2.4)	298.5 (2.0)	69.0 (0.7)
50	400	400	2.9 (0.1)	130.4 (1.2)	149.0 (2.4)	298.5 (2.0)	69.0 (0.7)
50	300	500	2.9 (0.1)	130.4 (1.2)	149.0 (2.4)	298.5 (2.0)	69.0 (0.7)
60	700	100	3.2 (0.1)	129.2 (1.2)	142.7 (2.3)	314.4 (2.2)	70.5 (0.7)
60	600	200	3.2 (0.1)	129.1 (1.3)	142.8 (2.3)	314.5 (2.2)	70.6 (0.7)
60	500	300	3.2 (0.1)	129.1 (1.3)	142.8 (2.3)	314.5 (2.2)	70.6 (0.7)
60	400	400	3.2 (0.1)	129.1 (1.3)	142.8 (2.3)	314.5 (2.2)	70.6 (0.7)
60	300	500	3.2 (0.1)	129.1 (1.3)	142.8 (2.3)	314.5 (2.2)	70.6 (0.7)

Abbreviations: SE, standard error; SNAP, Supplemental Nutrition Assistance Program.

**Table 3.** Nutritional Intake of a Family of 4 by Funding and Time Availability When SNAP Funds Can Be Used to Purchase Deli Foods

Time, min/d	SNAP funding, $/mo	Self-funding, $/mo	No. of servings of fruits/vegetables per day per person, mean (SE)	Recommended consumption, mean (SE), %			
				Protein	Sodium	Sugar	Fiber
20	700	100	1.8 (0.1)	124.2 (1.4)	171.5 (2.3)	270.5 (2.3)	67.1 (0.7)
20	600	200	1.8 (0.1)	124.1 (1.4)	171.1 (2.3)	270.4 (2.3)	67.1 (0.7)
20	500	300	1.8 (0.1)	124.1 (1.4)	171.0 (2.3)	270.4 (2.3)	67.1 (0.7)
20	400	400	1.8 (0.1)	124.1 (1.4)	171.1 (2.3)	270.4 (2.3)	67.1 (0.7)
20	300	500	1.8 (0.1)	124.1 (1.4)	171.1 (2.3)	270.4 (2.3)	67.1 (0.7)
30	700	100	2.3 (0.1)	125.4 (1.2)	157.2 (2.4)	276.2 (1.8)	67.4 (0.6)
30	600	200	2.3 (0.1)	125.4 (1.2)	157.2 (2.4)	276.2 (1.8)	67.4 (0.6)
30	500	300	2.3 (0.1)	125.3 (1.2)	157.2 (2.4)	276.2 (1.8)	67.4 (0.6)
30	400	400	2.3 (0.1)	125.4 (1.2)	157.2 (2.4)	276.2 (1.8)	67.4 (0.6)
30	300	500	2.3 (0.1)	125.4 (1.2)	157.2 (2.4)	276.2 (1.8)	67.4 (0.6)
40	700	100	2.7 (0.1)	126.2 (1.2)	151.6 (2.6)	287.0 (1.7)	67.9 (0.7)
40	600	200	2.7 (0.1)	126.2 (1.2)	151.5 (2.6)	286.9 (1.7)	67.8 (0.7)
40	500	300	2.7 (0.1)	126.2 (1.2)	151.7 (2.6)	287.0 (1.7)	67.9 (0.7)
40	400	400	2.7 (0.1)	126.2 (1.2)	151.6 (2.6)	287.0 (1.7)	67.9 (0.7)
40	300	500	2.7 (0.1)	126.2 (1.2)	151.6 (2.6)	287.0 (1.7)	67.9 (0.7)
50	700	100	3.0 (0.1)	126.8 (1.3)	144.3 (2.6)	297.8 (1.9)	68.5 (0.7)
50	600	200	3.0 (0.1)	126.8 (1.3)	144.3 (2.6)	297.8 (1.9)	68.5 (0.7)
50	500	300	3.0 (0.1)	126.8 (1.3)	144.3 (2.6)	297.8 (1.9)	68.5 (0.7)
50	400	400	3.0 (0.1)	126.8 (1.3)	144.3 (2.6)	297.8 (1.9)	68.5 (0.7)
50	300	500	3.0 (0.1)	126.8 (1.3)	144.3 (2.6)	297.8 (1.9)	68.5 (0.7)
60	700	100	3.4 (0.1)	126.6 (1.3)	139.9 (2.5)	310.9 (1.9)	70.7 (0.7)
60	600	200	3.4 (0.1)	126.6 (1.3)	139.9 (2.5)	310.9 (1.9)	70.7 (0.7)
60	500	300	3.4 (0.1)	126.6 (1.3)	139.9 (2.5)	310.9 (1.9)	70.7 (0.7)
60	400	400	3.4 (0.1)	126.6 (1.3)	139.9 (2.5)	310.9 (1.9)	70.7 (0.7)
60	300	500	3.4 (0.1)	126.6 (1.3)	139.9 (2.5)	310.9 (1.9)	70.7 (0.7)

Abbreviations: SE, standard error; SNAP, Supplemental Nutrition Assistance Program.

We also explored the potential effect of disallowing the use of SNAP funds for frozen prepared foods. This scenario is similar although not identical to proposals in which foods from a specific list are allowable for purchase with SNAP funds.^20^ This restriction is because the effect of allowing only foods from a specific list of categories is in effect similar to disallowing specific categories of food. Table 4 provides simulation results for our model under SNAP rules that disallow spending on frozen prepared foods and allow spending on deli foods. Comparing these results with those in Tables 2 and 3 shows that disallowing spending on frozen prepared foods was not associated with improved nutrition. For example, in the scenario with 40 min/d of cooking time, $400/mo of SNAP benefits, and $400/mo of self-funding, the meal plan for which SNAP funds can be spent on deli foods but not prepared frozen foods had a mean (SE) of 2.6 (0.1) servings/d per person of fruits/vegetables, 127.5% (1.3%) of daily recommended protein per person, 153.3% (2.6%) of daily recommended sodium per person, 284.8% (1.8%) of daily recommended sugar per person, and 68.3% (0.6%) of daily recommended fiber per person.

**Table 4.** Nutritional Intake of a Family of 4 by Funding and Time Availability When SNAP Funds Can Be Used to Purchase Deli Foods But Not Prepared Frozen Foods

Time, min/d	SNAP funding, $/mo	Self-funding, $/mo	No. of servings of fruits/vegetables per day per person, mean (SE)	Recommended consumption, mean (SE), %			
				Protein	Sodium	Sugar	Fiber
20	700	100	1.1 (<0.1)	129.6 (1.4)	160.1 (2.6)	308.4 (1.4)	67.5 (1.0)
20	600	200	1.2 (0.1)	143.2 (2.0)	181.9 (2.7)	283.8 (2.8)	64.3 (1.0)
20	500	300	1.5 (0.1)	134.0 (2.0)	178.2 (2.5)	274.4 (2.7)	65.5 (1.1)
20	400	400	1.7 (<0.1)	126.8 (1.6)	177.8 (2.4)	265.5 (2.1)	66.9 (0.8)
20	300	500	1.7 (<0.1)	124.4 (1.4)	174.1 (2.5)	265.6 (2.0)	67.7 (0.7)
30	700	100	1.8 (0.1)	141.9 (2.3)	153.4 (2.8)	270.7 (3.1)	58.0 (1.2)
30	600	200	2.2 (0.1)	135.2 (1.8)	162.0 (2.8)	269.7 (2.6)	62.8 (1.1)
30	500	300	2.3 (0.1)	130.1 (1.5)	164.8 (2.6)	269.7 (2.0)	65.5 (0.8)
30	400	400	2.2 (0.1)	127.6 (1.3)	161.2 (2.6)	273.1 (1.8)	67.7 (0.5)
30	300	500	2.2 (0.1)	126.8 (1.2)	163.4 (2.7)	274.2 (1.6)	68.4 (0.6)
40	700	100	2.6 (0.1)	137.0 (1.8)	141.4 (3.2)	267.6 (2.7)	59.0 (1.1)
40	600	200	2.8 (0.1)	133.3 (1.7)	148.1 (2.5)	266.2 (1.8)	62.7 (0.9)
40	500	300	2.7 (0.1)	130.5 (1.4)	153.6 (2.7)	276.2 (1.8)	66.5 (0.7)
40	400	400	2.6 (0.1)	127.5 (1.3)	153.3 (2.6)	284.8 (1.8)	68.3 (0.6)
40	300	500	2.6 (0.1)	127.3 (1.2)	154.5 (2.7)	285.5 (1.8)	68.6 (0.6)
50	700	100	3.2 (0.1)	134.9 (1.7)	130.4 (2.5)	271.8 (2.0)	61.1 (1.0)
50	600	200	3.1 (0.1)	133.0 (1.5)	141.8 (2.5)	281.1 (1.7)	65.0 (0.8)
50	500	300	3.0 (0.1)	129.4 (1.3)	149.0 (2.7)	293.9 (1.8)	68.7 (0.7)
50	400	400	2.9 (0.1)	127.6 (1.2)	150.7 (2.7)	300.0 (2.0)	70.4 (0.6)
50	300	500	2.9 (0.1)	127.7 (1.2)	151.6 (2.7)	300.4 (2.0)	70.5 (0.6)
60	700	100	3.5 (0.1)	134.8 (1.7)	124.1 (2.8)	287.9 (1.9)	63.1 (0.8)
60	600	200	3.5 (0.1)	130.6 (1.4)	134.8 (2.3)	300.4 (2.0)	67.2 (0.7)
60	500	300	3.4 (0.1)	127.5 (1.2)	142.8 (2.5)	310.8 (1.8)	71.4 (0.7)
60	400	400	3.3 (0.1)	127.1 (1.1)	144.6 (2.6)	316.1 (2.0)	72.3 (0.6)
60	300	500	3.3 (0.1)	127.3 (1.1)	144.7 (2.6)	315.6 (2.0)	72.3 (0.6)

Abbreviations: SE, standard error; SNAP, Supplemental Nutrition Assistance Program.

## Discussion

Our model simulation results (Figure, A) suggest that allotting SNAP recipients more time and less money for meal preparation is associated with healthier home-cooked meals. The association between greater time availability and the nutritional benefits of more home-cooked meals can be observed through reduced sodium consumption (Figure, D) and increased consumption of fruits and vegetables (Figure, B). Although additional funding was associated with purchasing more frozen foods, the model also found an association between this additional funding and the purchase of additional snacks, especially fruits and vegetables. Moreover, some foods with higher fiber content (such as whole grain foods) have higher costs. These factors provide mechanisms to help explain the association of increased consumption of fiber (Figure, F) and fruits and vegetables (Figure, B) and decreased percentage of home-cooked meals (Figure, A) with increased household spending. At all levels of spending and time availability, SNAP recipients maintained levels of protein consumption at or slightly above the recommended levels (Figure, C). However, sugar (Figure, E) and sodium (Figure, D) consumption were always substantially above the recommended levels. Greater household spending was associated with increases in sugar and sodium consumption due in part to the high sugar and sodium content of many frozen and restaurant-bought foods. Interestingly, greater time availability was also associated with increased sugar consumption—which, according to the model, may be due to increased consumption of sugar-heavy, homemade breakfast foods.

Although previous studies^57,58^ have reported associations between time availability and the proportion of home-cooked meals, they did not evaluate whether increased SNAP funding might compensate for a lack of time availability. Our analysis found that time availability and funding are not strict substitutes for each other. Although increased funding was associated with improved nutritional quality in some respects (namely, consumption of fruits, vegetables, and fiber), it was associated with decreased nutritional quality in others (namely, sodium and sugar consumption). On the other hand, increased time availability was associated with increases in the percentage of home-cooked meals, further increases in the consumption of fruits and vegetables, and decreases in sodium consumption. However, increased time availability was also associated with increased sugar consumption.

The results of our model simulations when SNAP funds are allowed to be used to purchase deli foods show that deli foods partially replace frozen prepared foods. Many frozen prepared foods are high in sodium or sugar, which is linked to obesity and cardiovascular disease.^53,57,59,60^ This modification targets SNAP recipients who have little time for cooking. Instead of being forced to depend on frozen prepared foods with high levels of sodium, recipients may instead buy time with these more nutritious prepared meals. Our results show that although this strategy can decrease overall spending on frozen prepared foods, it does not appreciably adjust nutritional trends. Disallowing the purchase of prepared frozen foods also was not associated with improved nutrition.

### Limitations

There are some limitations to our study. Two limitations are related to how we consider dietary preferences. First, we modeled the dietary preference of a food by summing the preference score of its constituent ingredients. This method is a useful approximation, but there are some foods for which the preference of the food may be higher than that of its individual ingredients. Second, we did not consider the potential association between the meal preferences of SNAP recipients and the dietary choices of their coworkers and friends. Another 2 limitations are related to how we consider time availability. First, our model cannot account for mismatches between meal preparation time and the time budget (eg, those arising from longer or shorter meal preparation times due to family-specific factors such as cooking speed) because we use as data the corresponding meal preparation time taken from the recipe for that meal. Second, living in a neighborhood without access to healthy foods (a “food desert”) could increase the amount of time required to prepare meals in a way that is not considered in our model; however, the literature suggests only a weak association between food deserts and dietary intake.^61^ Moreover, we believe that our proposal of governmental interventions that increase time availability would only be more beneficial for families without access to healthy foods. Additional limitations are that we did not allow for ingredient substitutions in recipes and required that any particular recipe or prepared food was not consumed more than 5 times a month, although these limitations are mitigated by the fact that we included 3327 recipes and 118 prepared foods in our model. A final limitation is that we could not ensure a representative set of recipes for the entire US owing to regional variations in meal preferences, although we attempted to select recipes that we perceived to be popular throughout the country.

## Conclusions

Lower-income families are more likely to either always cook at home or never cook at home, with Black households the least likely to cook at home and immigrant households the most likely.^62^ Work schedules, commute distance, family composition, and local availability of food are factors that affect how much time families invest in cooking, and lower-income families in many neighborhoods are more likely to work flexible hours, have longer commute distances, and have less local availability of food. Families with younger children may have less time availability for cooking,^63^ whereas families with older children may have more family members who are able to assist in cooking. In this study, the addition of deli foods among the list of SNAP-eligible foods was insufficient to shift the nutritional profile of SNAP recipients. Similarly, the removal of prepared frozen foods from the list of SNAP-eligible foods was ineffective in improving nutrition. Instead, we recommend further development and marketing by food labels of prepared meals with higher levels of nutritional components that can make healthy and affordable diets achievable for families with limited financial and temporal means.

With the current food supply; overabundance of calorically dense, inexpensive foods; and high cost of nutritious foods, it is critical that foods are made available for purchase in line with the Dietary Guidelines for Americans.^64^ Future research could model the influence of nutritious, inexpensive, ready-to-prepare meals in a hypothetical scenario in which they are widely available, in which case increased funding could assist time-constrained families. However, an abundance of such meals is not currently available. The results of this decision analytical model suggest that governmental interventions that increase meal preparation time availability may improve nutritional outcomes for SNAP participants. Rather than increasing SNAP funding for purchasing of food, an alternative is to provide funding specifically for services that allow SNAP participants to buy time. Research on governmental interventions that increase meal preparation time availability for low-income households in low-income neighborhoods is currently limited; therefore, further study of funding for childcare, housecleaning services, grocery delivery, or healthy meal kit deliveries to increase time availability is warranted.

## Notes

Supplement. **eTable.** Nutritional Intake of a Typical Family of 4 Under Different Financial and Time Availability Scenarios Under Existing SNAP Policies **eMethods.** Mathematical Formulation, Data, and Model Calibration eReferences
