# Supplementary material for: Association of Funding and Meal Preparation Time With Nutritional Quality of Meals of Supplemental Nutritional Assistance Program Recipients
Source: JAMA Netw Open. 2021 Jun 24;4(6):e2114701. doi: 10.1001/jamanetworkopen.2021.14701 (PMC8226420; doi:10.1001/jamanetworkopen.2021.14701)
Supplement: Supplement. — eTable. Nutritional Intake of a Typical Family of 4 Under Different Financial and Time Availability Scenarios Under Existing SNAP Policies eMethods. Mathematical Formulation, Data, and Model Calibration eReferences [file jamanetwopen-e2114701-s001.pdf]

Supplementary Online Content

Olfat M, Laraia BA, Aswani AJ. Association of funding and meal preparation time with nutritional quality of meals of Supplemental Nutritional Assistance Program recipients. *JAMA Netw Open*. 2021;4(6):e2114701. doi:10.1001/jamanetworkopen.2021.14701

eTable. Nutritional Intake of a Typical Family of 4 Under Different Financial and Time Availability Scenarios Under Existing SNAP Policies

eMethods. Mathematical Formulation, Data, and Model Calibration

eReferences

This supplementary material has been provided by the authors to give readers additional information about their work.

© 2021 Olfat M et al. *JAMA Network Open*.

eTable. Nutritional Intake of a Typical Family of 4 Under Different Financial and Time Availability Scenarios Under Existing SNAP Policies

Time (min per day)	SNAP ($ per month)	Self ($ per month)	Home-Cooked Meals (%)	Fruits-Vegetables (servings per day per person)	Protein (% of recommendation)	Sodium (% of recommendation)	Sugar (% of recommendation)	Fiber (% of recommendation)
20	400	100	20.1% (0.3%)	0.5 (<0.1)	100.3% (0.6%)	115.1% (0.8%)	241.8% (1.0%)	31.2% (0.3%)
20	400	200	15.5% (0.6%)	0.8 (<0.1)	113.8% (0.9%)	113.4% (1.1%)	251.2% (1.2%)	40.6% (0.4%)
20	400	300	16.6% (0.9%)	1.4 (<0.1)	116.8% (1.0%)	146.2% (2.0%)	259.2% (1.5%)	52.4% (0.6%)
20	400	400	17.6% (0.9%)	1.8 (<0.1)	122.7% (1.3%)	167.3% (2.1%)	266.6% (1.9%)	66.5% (0.7%)
20	400	500	21.7% (1.0%)	2.3 (0.1)	127.5% (1.5%)	182.0% (2.7%)	279.9% (2.7%)	78.1% (0.8%)
20	400	600	23.9% (1.0%)	2.8 (0.1)	134.9% (1.6%)	200.9% (3.1%)	295.1% (3.1%)	90.1% (1.0%)
30	400	100	31.2% (0.6%)	0.7 (<0.1)	104.0% (0.8%)	114.5% (0.9%)	252.2% (1.5%)	31.9% (0.3%)
30	400	200	24.7% (1.0%)	1.2 (<0.1)	112.4% (0.9%)	125.2% (1.2%)	264.1% (1.5%)	41.9% (0.4%)
30	400	300	24.6% (1.1%)	1.8 (<0.1)	119.5% (1.0%)	138.5% (1.8%)	268.7% (2.0%)	53.8% (0.6%)
30	400	400	24.5% (1.1%)	2.3 (<0.1)	125.9% (1.1%)	154.4% (2.3%)	274.6% (2.1%)	66.0% (0.7%)
30	400	500	26.3% (1.1%)	2.8 (0.1)	132.6% (1.6%)	170.4% (2.8%)	281.8% (2.4%)	77.8% (0.9%)
30	400	600	28.9% (1.1%)	3.2 (0.1)	139.1% (1.8%)	185.9% (3.1%)	295.9% (2.9%)	89.1% (0.9%)
40	400	100	38.6% (0.9%)	1.0 (<0.1)	105.1% (0.9%)	111.7% (1.0%)	267.2% (1.5%)	34.3% (0.4%)
40	400	200	32.5% (1.2%)	1.5 (<0.1)	112.6% (0.9%)	122.9% (1.4%)	277.4% (2.0%)	44.4% (0.4%)
40	400	300	32.3% (1.1%)	2.2 (<0.1)	118.4% (1.0%)	132.9% (1.8%)	280.9% (2.0%)	55.6% (0.6%)
40	400	400	31.5% (1.3%)	2.7 (0.1)	126.6% (1.1%)	146.3% (2.3%)	286.1% (2.0%)	67.0% (0.7%)
40	400	500	31.2% (1.3%)	3.2 (0.1)	133.8% (1.4%)	163.3% (2.7%)	292.5% (2.2%)	79.1% (0.8%)
40	400	600	33.0% (1.2%)	3.6 (0.1)	141.6% (1.7%)	177.9% (3.3%)	303.4% (2.9%)	89.7% (0.9%)
50	400	100	45.8% (0.9%)	1.3 (<0.1)	105.8% (1.0%)	111.6% (1.0%)	283.7% (1.5%)	36.7% (0.4%)
50	400	200	40.8% (1.2%)	1.9 (<0.1)	111.9% (1.0%)	117.3% (1.3%)	292.8% (2.1%)	46.6% (0.5%)
50	400	300	38.1% (1.2%)	2.5 (<0.1)	118.9% (1.1%)	128.4% (1.8%)	295.2% (2.1%)	57.3% (0.6%)
50	400	400	36.2% (1.3%)	3.0 (0.1)	126.7% (1.1%)	141.4% (2.3%)	299.6% (2.0%)	68.5% (0.6%)
50	400	500	36.4% (1.3%)	3.5 (0.1)	135.2% (1.3%)	156.3% (2.5%)	304.6% (2.1%)	79.8% (0.8%)
50	400	600	38.2% (1.2%)	4.0 (0.1)	144.2% (1.8%)	171.4% (3.0%)	312.0% (2.5%)	90.3% (1.0%)
60	400	100	52.7% (0.9%)	1.4 (<0.1)	109.0% (1.1%)	108.7% (1.0%)	298.6% (2.0%)	38.8% (0.4%)
60	400	200	48.3% (1.1%)	2.1 (<0.1)	112.5% (1.0%)	117.0% (1.3%)	307.5% (2.2%)	48.8% (0.5%)
60	400	300	45.4% (1.2%)	2.8 (<0.1)	119.5% (1.0%)	124.4% (1.7%)	309.0% (2.1%)	59.0% (0.6%)
60	400	400	43.0% (1.2%)	3.4 (0.1)	126.2% (1.1%)	134.8% (2.2%)	313.1% (2.3%)	69.8% (0.6%)

© 2021 Olfat M et al. *JAMA Network Open*.

60	400	500	41.1% (1.3%)	3.8 (0.1)	136.0% (1.3%)	151.4% (2.6%)	318.9% (2.1%)	81.3% (0.8%)
60	400	600	42.8% (1.2%)	4.3 (0.1)	144.4% (1.8%)	165.2% (2.8%)	322.4% (2.4%)	91.0% (0.9%)

© 2021 Olfat M et al. *JAMA Network Open*.

eMethods. Mathematical Formulation, Data, and Model Calibration

Mathematical Formulation

Our model chooses a meal plan that best matches the dietary preferences of an average SNAP participant. This is modeled by assigning to each ingredient an ingredient-preference score, assigning a recipe-preference score by summing the ingredient-preference scores of the constituent ingredients, and then choosing a meal plan that maximizes the sum of the recipe-preference scores of the selected recipes in the meal plan. Our model maximizes the recipe-preference scores of the selected meal plan to more accurately model consumption-behavior rather than modeling purchasing-behaviors. The optimization software used with our model to generate simulation results is Gurobi version 9.1.0[1], and its mathematical formulation is below:

$$\max \sum_{j=1}^{n} u_j y_j$$

$$(1), s.t. \sum_{i=1}^{m} c_i x_i \leq C_1 + C_2$$

$$(2), \quad \sum_{i=1}^{m} (1 - e_i) c_i x_i \leq C_2$$

$$(3), \quad \sum_{j=1}^{n} t_j y_j \leq 30T$$

$$(4), \quad \sum_{i=1}^{m} a_{ij} x_i \geq y_j, \qquad j = 1, \dots, n$$

$$(5), \quad y_j \leq 5D_j, \qquad j = 1, \dots, n$$

$$(6), \quad \sum_{i=1}^{n} m_i y_i \geq 240$$

$$(7), \quad \sum_{i=1}^{n} b_i y_i \geq 40$$

$$(8), \quad \sum_{i=1}^{n} d_i y_i \geq 120$$

$$(9), \quad x_i \in \mathbb{Z}_+, \qquad i = 1, \dots, m$$

$$(10), \quad y_j \in \mathbb{Z}_+, \qquad j = 1, \dots, n$$

© 2021 Olfat M et al. *JAMA Network Open*.

The number of ingredients is m, and the number of recipes is n. Here, x_i is the number of units of ingredient i purchased, and y_j is the number of units of recipe j cooked. (We treat pre-prepared foods as recipes with zero cooking time.) The quantity a_{ij} is the number of units of ingredient i required for one unit of recipe j. The quantity D_j denotes the number of servings resulting from making recipe j once. The value c_i is the cost of one unit of ingredient i. The values C_1 and C_2 denote the SNAP benefits and household funds available for purchasing food, respectively, and they are model inputs chosen by us. For an ingredient i, e_i takes value 1 if i is eligible for SNAP and 0 otherwise; this allows the model to specify which food groups are eligible for purchase with SNAP funds[2]. The quantity t_j represents the time required to prepare recipe j. The T is the average daily time budget, which is also a model input set by us.

For a recipe j, the quantity u_j represents the recipe-preference score of that recipe, which is computed by summing the ingredient-preference scores of the constituent ingredients. We add a small random component to the recipe-preference score to capture inter-family variations in preference; thus, two simulations of the model with identical inputs will return slightly different meal plans. We interpret any single simulation of the model as the food choices made by a randomly chosen family with ingredient-preferences close to, but not equivalent to, the typical SNAP participant. Specifically, in each run of a simulation we incorporate a recipe-preference multiplier for each recipe that is uniformly distributed between 0.9 and 1.1. We conduct 100 simulations for any given budget, and we report the mean and standard error.

In our model, equation (1) says the total cost of purchased ingredients is less than the total funds available, and equation (2) says the total cost of SNAP-ineligible ingredients is less than the amount of unrestricted personal funds. Equation (3) says the total time spent preparing meals is less than the total time budget. Equation (4) says the quantity of purchased ingredients is sufficient to cook the recipes in the meal plan. Equation (5) says that no meal is consumed more than 5 times in a month per family member. For beverages and salty snacks, this limit is doubled. Furthermore, we assume that beneficiaries will attempt to procure 60 meals (30 lunches and 30 dinners) for each family member, as well as breakfast for at least 20 days, before spending on desserts and other auxiliary foods (as opposed to purchasing mainly snacks). The model requires less breakfast consumption because studies show that over half of Americans under 65 do not regularly eat breakfast, with 25% of adults reporting that

© 2021 Olfat M et al. *JAMA Network Open*.

they rarely or never eat breakfast[3]. The total lunch and dinner count is enforced in equation (6), and the breakfast count is enforced in equation (7). We also distinguish between lunch and dinner, assuming dinners to be meals with over 600 calories per serving; in equation (8), we enforce that each family member consume at least one dinner-sized meal per day. Finally, equations (9) and (10) require that variables x_i and y_j be integer-valued to represent that whole (i.e., non-fractional) quantities of ingredients are purchased and whole quantities of recipes are prepared.

Data

The data used in our model was accessed in February 2017. The recipes in our model are from the British Broadcasting Corporation Food's online database of 11,026 recipes[4]. Each recipe included a name, a list of ingredients and amounts, dietary restrictions, and approximate preparation time. We removed recipes for dishes uncommonly eaten in the United States (n=5890) by eliminating recipes using meats or vegetables (e.g., turbot and free-range quail) not easily found at a typical grocery story. Safeway Inc.'s online site for groceries was used as a proxy for what is "reasonable" to find at a typical grocery store. We also removed recipes with preparation times greater than 45 minutes (n=1671), as these are unlikely for use by time-constrained beneficiaries. A small percentage of recipes also exhibited inconsistencies in how ingredient amounts were recorded (n=138), and so these recipes were removed as well. Overall, this left 3,327 recipes for use in our model.

This list included recipes for both meals and desserts, which were labeled accordingly. For this study, sugary recipes, such as tarts or cakes, were classified as desserts. Recipes with larger carbohydrate components (e.g., rice or bread) or with a focus on vegetables (e.g., broccoli), were classified as meals. Within this, meals that accounted for over 600 calories per serving were classified as dinner meals. Finally, any recipe with "eggs", "toast", "waffles", "pancakes", "scones" or "breakfast" in the name (meal or not) was classified as breakfast. For each recipe, we define the label $m_j = 1$ if recipe j is a meal and 0 otherwise, $b_j = 1$ if j is breakfast and 0 otherwise, and $d_j = 1$ if j is a dinner meal and 0 otherwise.

Additional food items from Safeway, Inc. were added to our model. A representative sample of the Safeway inventory of frozen prepared foods, snack foods, beverages, cereals, individual ingredients, and deli items

© 2021 Olfat M et al. *JAMA Network Open*.

were added. Since this analysis focuses on time, foods from several top fast-food restaurants were also added. For fast-foods, prepared foods, frozen prepared foods, beverages, and snacks, including fruits and vegetables eaten as snacks, each individual unit is recorded as a "recipe" with only one ingredient. For single-item foods that may be purchased in single-serving denominations (e.g., fruits and vegetables), denominations were chosen as to comprise one serving size. For frozen prepared foods and beverages, different purchase denominations were included (e.g., single-serving and family-size prepared meals). In the model, we assume that all family members get the same number of servings. We set D_j to 4 for prepared foods, frozen prepared foods, beverages, and fruits and vegetables eaten as snacks, since these foods may be purchased in any denomination and we assume a family of 4.

Prices and amounts for the purchase of individual ingredients were drawn from Safeway, Inc.'s online shopping site for the ZIP code 94702 (Berkeley, CA)[5]. As the second-largest supermarket chain in the US with 1,678 stores in 18 states, we believed Safeway to provide an accurate estimate of the relative prices, product sizes, and availability of groceries throughout the Unites States. Our estimate was taken for the Berkeley area, but the results of our simulation analysis can be done for other areas in the country by incorporating multipliers for inflation and geographic location[6]. Generally, purchase denominations for ingredients were chosen as the smallest possible. Prices for fast foods were drawn from the Fast Food Menu Prices website[7].

Dietary preferences were determined using published literature on the preferences of SNAP participants, specifically by bucketing ingredients into one of 30 primary groups[8]. To weight food items by preference, each ingredient group was assigned an ingredient-preference score, ranging from 1 to 30 with the most-preferred ingredient group being assigned a 30 and the least-preferred ingredient group being assigned a 1. Last, nutritional information for all ingredients was taken from the USDA's online Food Composition Database[9].

Model Calibration

To ensure that our model accurately captures observed preferences, we compared the predicted meal plans to behaviors described in the literature. An American Time Use Survey (ATUS) in 2008 found that only 41.7% of men and 67.7% of women were likely to cook on any given day, and that they cooked for 45 minutes and 65.6 minutes on average on days that they did cook, respectively[10]. Furthermore, a recent report stated that the

© 2021 Olfat M et al. *JAMA Network Open*.

median per-person spending on food for American households in 2015 was $50 per-day[11]. Recent studies also suggest that the average American consumes about 3400 milligrams of sodium, 80 grams of added sugars, 90 grams of protein, 16 grams of fiber, and 2.5 servings of fruits and vegetables per day[12–15]. The model was run 100 times with parameters of 40 minutes per day of cooking time and $800 of food expenditures (all assumed unrestricted) and attained an average daily per-person consumption of 3528 milligrams of sodium, 87 grams of sugars, 89 grams of protein, 21 grams of fiber, and 2.6 servings of fruits and vegetables. As the goal of our study is to study directional effects of policy and time, we consider this to be verification of our model's validity.

eReferences

1. Gurobi Optimization LLC. *Gurobi Optimizer Reference Manual.*; 2021. http://www.gurobi.com

2. *A Quick Guide to SNAP Eligibility and Benefits*. Center on Budget and Policy Priorities (CBPP); 2017.

3. Haines PS, Guilkey DK, POPKIN B. Trends in breakfast consumption if US adults between 1965 and 1991. *Journal of the American Dietetic Association*. 1996;96(5):464-470.

4. BBC Food - Recipes. Accessed April 8, 2017. https://www.bbc.co.uk/food/recipes

5. Safeway Grocery Delivery. Accessed April 8, 2017. http://www.safeway.com/

6. Gottlober P, Brady T, Robinson B, Davis T, Phillips S, Gruber A. *Medicare Hospital Prospective Payment System: How DRG Rates Are Calculated and Updated*. Centers for Medicare & Medicaid Services; 2001.

7. Fast Food Menu Prices. Accessed April 8, 2017. https://www.fastfoodmenuprices.com/

8. Garasky S, Mbwana K, Romualdo A, Tenaglio A, Roy M. *Foods Typically Purchased by Supplemental Nutrition Assistance Program (SNAP) Households*. U.S. Department of Agriculture, Food and Nutrition Service; 2016.

9. *Food Composition Database*. U.S. Department of Agriculture; 2017.

10. Smith LP, Ng SW, Popkin BM. Trends in US home food preparation and consumption: analysis of national nutrition surveys and time use studies from 1965–1966 to 2007–2008. *Nutrition journal*. 2013;12(1):45.

11. Coleman-Jensen A, Gregory C, Singh A. *Household Food Security in the United States in 2013*. U.S. Department of Agriculture, Economic Research Service; 2014.

12. Rehm CD, Peñalvo JL, Afshin A, Mozaffarian D. Dietary intake among US adults, 1999-2012. *Jama*. 2016;315(23):2542-2553.

13. King DE, Mainous III AG, Lambourne CA. Trends in dietary fiber intake in the United States, 1999-2008. *Journal of the Academy of Nutrition and Dietetics*. 2012;112(5):642-648.

© 2021 Olfat M et al. *JAMA Network Open*.

14. Welsh JA, Sharma AJ, Grellinger L, Vos MB. Consumption of added sugars is decreasing in the United States–. *The American journal of clinical nutrition*. 2011;94(3):726-734.

15. Fulgoni III VL. Current protein intake in America: analysis of the National Health and Nutrition Examination Survey, 2003–2004. *The American journal of clinical nutrition*. 2008;87(5):1554S-1557S.

© 2021 Olfat M et al. *JAMA Network Open*.
